# The Association between Patient Characteristics and Biochemical Recurrence after Radical Prostatectomy

**DOI:** 10.3390/medicina60071119

**Published:** 2024-07-11

**Authors:** Carolin Siech, Mike Wenzel, Carsten Lange, Cristina Cano Garcia, Clara Humke, Zhe Tian, Pierre I. Karakiewicz, Miriam Traumann, Luis A. Kluth, Felix K. H. Chun, Benedikt Hoeh, Philipp Mandel

**Affiliations:** 1 Department of Urology, University Hospital, Goethe University Frankfurt, 60590 Frankfurt am Main, Germany; 2 Cancer Prognostics and Health Outcomes Unit, Division of Urology, University of Montréal Health Center, Montréal, QC H2X 3E4, Canada

**Keywords:** age, BCR, body mass index, prostate cancer, prostate volume, radical prostatectomy

## Abstract

*Background*: Biochemical recurrence (BCR) represents the rise of prostate-specific antigen (PSA) levels after treatment with curative radical prostatectomy (RP) or radiation for prostate cancer. The objective of the current study was to test for the association between patient characteristics, namely age, body mass index (BMI), as well as prostate volume at surgery, and BCR after RP. *Material and Methods*: Within a tertiary care database, patients with prostate cancer treated with RP between January 2014 and June 2023 were included. Kaplan–Meier survival analyses and Cox regression models addressed BCR after RP according to patient characteristics. *Results*: Of 821 patients, the median age was 66 years (interquartile range [IQR] 61–71 years), BMI was 26.2 kg/m^2^ (IQR 24.3–28.8 kg/m^2^), and prostate volume was 40 cm^3^ (IQR 30–55 cm^3^). Median follow-up was 20 months. In survival analyses, the three-year BCR-free survival rates were 81 vs. 84 vs. 81% in patients aged ≤60 vs. 61–69 vs. 70 years (*p* = 0.1). In patients with BMI < 25.0 vs. 25.0–29.9 vs. ≥30.0 kg/m^2^, the three-year BCR-free survival rates were 84 vs. 81 vs. 84% (*p* = 0.7). In patients with prostate volume ≤40 vs. >40 cm^3^, the three-year BCR-free survival rates were 85 vs. 80% (*p* = 0.004). In multivariable Cox regression models accounting for patient and pathologic tumor characteristics and adjuvant radiation therapy, a higher prostate volume independently predicted BCR as continuous (hazard ratio 1.012, 95% confidence interval 1.005–1.019; *p* < 0.001), as well as categorized the variable based on the median (hazard ratio 1.66, 95% confidence interval 1.17–2.36; *p* = 0.005). Conversely, neither age nor BMI were significantly associated with BCR after RP. *Conclusions*: The higher prostate volume independently predicted BCR after RP, but not age or BMI at surgery. Consequently, patients with an elevated prostate volume should be considered for closer postoperative follow-up.

## 1. Introduction

Radical prostatectomy, the complete removal of the prostate gland and seminal vesicles, represents a well-established guideline-recommended curative treatment option in patients with clinically localized or locally advanced prostate cancer [1,2]. Depending on the preoperative clinical risk constellation, radical prostatectomy is performed with or without pelvic lymph node dissection. Besides the open surgical approach, robotic-assisted surgery has been gaining importance in recent years. Despite its curative intent, some patients may experience biochemical recurrence (BCR) following surgery [3,4,5]. BCR is defined based on guideline definitions as an initial serum prostate-specific antigen (PSA) value of ≥0.2 ng/mL, with a second confirmatory level of >0.2 ng/mL in follow-up after radical prostatectomy [4,6].

Several pathologic characteristics are linked to worse short- and long-term oncological outcomes. Specifically, the non-organ-confined pathologic tumor stage [7,8], Gleason Grade Group ≥ 4 [7,9,10,11], as well as positive surgical margins [3,5,11], represent predictors of less favorable BCR and mortality. However, the prognostic value of patient characteristics, such as age [11,12,13,14], body mass index (BMI) [15], and prostate volume [16,17,18,19,20,21] regarding oncological outcomes, such as BCR after radical prostatectomy, remain unclear.

We addressed this uncertainty and hypothesized that BCR rates after radical prostatectomy might differ according to patient characteristics, namely age, BMI, and prostate volume at time of surgery. To address this hypothesis, we relied on a contemporary cohort of prostate cancer patients treated with radical prostatectomy in a tertiary care referral center.

## 2. Materials and Methods

### 2.1. Study Population

**Figure 1:**
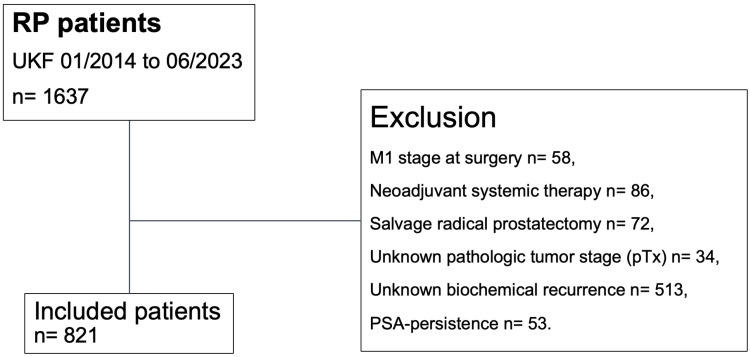
Consort diagram. Abbreviations: PSA= prostate-specific antigen; pT = pathologic tumor stage at surgery; RP = radical prostatectomy; UKF = University Hospital Frankfurt.

Within the prospectively maintained database of our tertiary care referral center, we retrospectively focused on patients with histologically confirmed adenocarcinoma of the prostate who were treated with either robotic-assisted or open retropubic radical prostatectomy between January 2014 and June 2023 (Figure 1). Based on the risk groups of the European Association of Urology (EAU), we included both localized as well as locally advanced prostate cancer patients [22]. Further inclusion criteria consisted of known follow-up regarding BCR and absence of prostate-specific antigen (PSA) persistence, defined as post-radical prostatectomy PSA of >0.1 ng/mL within six weeks postoperatively according to the EAU guidelines [22]. All patients with clinical suspicion of metastases at time of surgery (cM1), treatment with neoadjuvant systemic therapy (chemotherapy and/or hormonal therapy), previous radiation therapy of the prostate (salvage radical prostatectomy), and unknown pathologic tumor stage (pTx) were excluded. Informed written consent to participate in this study was given by all patients. Approval by the local ethics committee had been obtained prior to data collection. Reporting had been reviewed in accordance with the precepts established by the Helsinki Declaration.

### 2.2. Definition of Variables for Analyses

The primary endpoint of the study, namely BCR, was derived from patients’ self-reports in follow-up and was defined according to the EAU guidelines valid at the timepoint of BCR and the American Urological Association (AUA) guidelines as an initial serum PSA value of ≥0.2 ng/mL, with a second confirmatory level of >0.2 ng/mL in follow-up after radical prostatectomy [4,6]. Variables of interest represented patient characteristics, namely age, BMI, and prostate volume at surgery. All three variables of interest were initially considered as continuous variables in the analyses. Additionally, to gain deeper insight into the association between patient characteristics and BCR, each variable of interest was also categorized. Categorization of patient age was based on quartiles (≤60 vs. 61–69 vs. ≥70 years). BMI was calculated based on patients’ self-reports of weight and height and categorization of BMI was based on definition of healthy weight (<25.0 kg/m^2^), overweight (25.0–29.9 kg/m^2^), and obesity (≥30.0 kg/m^2^) by the World Health Organization (WHO) [23]. Preoperative prostate volume was measured via transrectal ultrasound (TRUS) performed by experienced urologists within our institution. Categorization of prostate volume was based on median (≤40 vs. >40 cm^3^), as was carried out in previous analyses [24,25].

### 2.3. Statistical Analyses

First, clinicopathological characteristics were tabulated. Medians and interquartile ranges were recorded for continuously coded variables. Frequencies and proportions were reported for categorical variables. Second, Kaplan–Meier plots depicted BCR-free survival rates according to patient characteristics, namely age, BMI, and prostate volume. Subsequently, univariable and multivariable Cox regression models addressed BCR according to patient characteristics. Adjustment variables consisted of preoperative PSA value, pathologic tumor stage (pTstage), Gleason Grade Group, pathologic lymph node stage (pNstage), positive surgical margin, and adjuvant radiation therapy. All tests were two sided, with a level of significance set at *p* < 0.05. R software environment was used for statistical computing and graphics (R version 4.3.2; R Foundation for Statistical Computing, Vienna, Austria) [26].

## 3. Results

### 3.1. Descriptive Characteristics

#### 3.1.1. Clinicopathological Characteristics

Within our institutional tertiary care database of 1637 prostate cancer patients who underwent radical prostatectomy between January 2014 and June 2023, we included 821 (50.2%) patients based on the above-described inclusion criteria (Figure 1). The median age was 66 years (interquartile range 61–71 years), the median BMI was 26.2 kg/m^2^ (interquartile range 24.3–28.8 kg/m^2^), and the median prostate volume was 40 cm^3^ (interquartile range 30–55 cm^3^). Further clinicopathological characteristics of the study cohort are summarized in Table 1.

#### 3.1.2. Kaplan–Meier Survival Analyses

**Figure 2:**
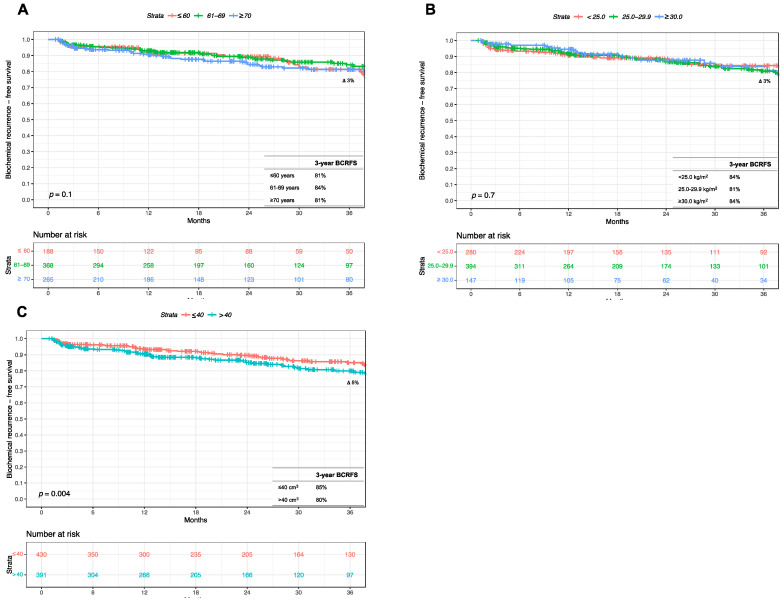
Kaplan–Meier survival analyses addressing biochemical recurrence (BCR)-free survival after radical prostatectomy (RP) according to (**A**) patient age (≤60 vs. 61–69 vs. ≥70 years), (**B**) body mass index (<25.0 vs. 25.0–29.9 vs. ≥30.0 kg/m^2^), and (**C**) prostate volume (≤40 vs. >40 cm^3^). Abbreviations: BCR = biochemical recurrence; BCRFS = biochemical recurrence-free survival; RP = radical prostatectomy.

At a median follow-up of 20 months (interquartile range 10–38 months), overall, 137 (17%) patients exhibited BCR. In Kaplan–Meier survival analyses, three-year BCR-free survival rates were 81 vs. 84 vs. 81% in patients aged ≤60 vs. 61–69 vs. ≥70 years, respectively (*p* = 0.1; Figure 2A). After stratification according to BMI categories, three-year BCR-free survival rates were, respectively, 84 vs. 81 vs. 84% in patients harboring BMI <25.0 vs. 25.0–29.9 vs. ≥30.0 kg/m^2^ (*p* = 0.7; Figure 2B). Finally, after stratification according to the median prostate volume, the three-year BCR-free survival rates were 85 vs. 80% in patients with preoperative prostate volume ≤40 vs. >40 cm^3^ (Δ 5%; *p* = 0.004; Figure 2C).

#### 3.1.3. Univariable and Multivariable Cox Regression Models

In univariable Cox regression models relying on continuously coded patient characteristics, a higher prostate volume predicted a higher BCR rate (hazard ratio 1.010, 95% confidence interval 1.004–1.016; *p* = 0.002; Table 2). Similarly, a prostate volume above the median (>40 cm^3^) predicted a higher BCR rate (hazard ratio 1.64, 95% confidence interval 1.17–2.31; *p* = 0.004). Even after adjustment for other baseline patient characteristics, preoperative PSA value, pathologic tumor characteristics, and adjuvant radiation therapy in multivariable Cox regression models, the continuously coded higher preoperative prostate volume (hazard ratio 1.012, 95% confidence interval 1.005–1.019; *p* < 0.001) as well as the categorized prostate volume above median (>40 cm^3^) remained independent predictors of a higher BCR rate after radical prostatectomy (hazard ratio 1.66, 95% confidence interval 1.17–2.36; *p* = 0.005). Conversely, neither age nor BMI were statistically significantly associated with BCR after radical prostatectomy in either univariable or multivariable Cox regression models.

## 4. Discussion

Within the current study, we hypothesized that patient characteristics, including age, BMI, and prostate volume may affect BCR rates after radical prostatectomy. Relying on a contemporary cohort of radical prostatectomy-treated prostate cancer patients at a tertiary care referral center between January 2014 and June 2023, we made several important observations.

First, we tabulated baseline patient characteristics of the study cohort. Within the contemporary cohort of 821 radical prostatectomy-treated prostate cancer patients, the median age was 66 years (interquartile range 61–71 years), the BMI was 26.2 kg/m^2^ (interquartile range 24.3–28.8 kg/m^2^), and prostate volume was 40 cm^3^ (interquartile range 30–55 cm^3^). The distribution of baseline patient characteristics, namely age, BMI, and prostate volume, recorded within the current study, is highly comparable to the distribution of patient characteristics of prostate cancer patients treated within other German tertiary care centers [13,17,18,27,28]. Thus, despite the limited sample size, the current study cohort appears eligible for subsequent survival analyses that address BCR as study endpoint.

Second, we did not identify statistically significant or clinically meaningful differences in BCR rates after radical prostatectomy according to patient age at surgery. Specifically, three-year BCR-free survival rates ranged from 81% in both ≤60-year-olds and ≥70-year-olds to 84% in 61–69-year-olds (*p* = 0.1). Similarly, after adjustment for other baseline patient characteristics (BMI and prostate volume), preoperative PSA value, pathologic tumor characteristics, and adjuvant radiation therapy in multivariable Cox regression models, patient age did not achieve independent predictor status for BCR rate after radical prostatectomy, either as a continuously coded variable (*p* = 0.5) or after stratification based on quartiles (*p* = 0.1 and *p* = 0.6). These observations confirm previous assessments by Leyh-Bannurah et al. and Tilki et al. in which patient age was not associated with BCR in multivariable models [13,14]. Conversely, historical analyses by Öbek et al. suggested a prognostic role of patient age for BCR after radical prostatectomy [12]. However, all three analyses categorized patient age and did not examine it as a continuous variable [12,13,14]. Moreover, Öbek et al. relied on a historical study cohort of North American patients who underwent radical retropubic prostatectomy between 1991 and 1998 [12]. In consequence, these differences in the study cohort selection render direct comparisons.

Third, we did not identify statistically significant differences in BCR rates after radical prostatectomy according to patient BMI nor between overweight (25.0–29.9 kg/m^2^) and obese (≥30.0 kg/m^2^) patients compared to their counterparts with a healthy weight (<25.0 kg/m^2^). Specifically, three-year BCR-free survival rates were 84 vs. 81 vs. 84% in patients harboring BMI <25.0 kg/m^2^ vs. 25.0–29.9 kg/m^2^ vs. ≥30.0 kg/m^2^ (*p* = 0.7). Similarly, in multivariable Cox regression models, neither continuously coded BMI (*p* = 0.8) or categorized BMI as overweight (25.0–29.9 kg/m^2^) or obesity (≥30.0 kg/m^2^) compared to healthy weight (<25.0 kg/m^2^) achieved independent predictor status for BCR rate after radical prostatectomy within the current study cohort (both *p* = 0.6). A contemporary systemic review and meta-analysis of 86,490 patients by Rivera-Izquierdo et al. reported a moderate relationship between obesity with BCR after radical prostatectomy, but not between overweight and BCR [15]. However, Rivera-Izquierdo et al. also reported a high heterogeneity between the included studies [15]. In particular, analyses relying on cut-offs different from the WHO definition of obesity as well as those without consideration of positive surgical margins as adjustment variables in multivariable models described a stronger association between obesity and BCR [29,30,31]. Consequently, these considerations might explain the differences between the observations recorded in the current study and those of previous reports.

Fourth, we addressed BCR rates after radical prostatectomy according to preoperative prostate volume. After stratification according to median prostate volume (40 cm^3^), three-year BCR-free survival rates were 85% in patients with a preoperative prostate volume ≤40 cm^3^ vs. 80% in patients with a preoperative prostate volume >40 cm^3^ (*p* = 0.004). Even after adjustment for other baseline patient characteristics (age and BMI), preoperative PSA value, pathologic tumor characteristics, and adjuvant radiation therapy in multivariable Cox regression models, the higher continuously coded preoperative prostate volume (hazard ratio 1.012; *p* < 0.001) as well as the categorized prostate volume above median (>40 cm^3^: hazard ratio 1.66; *p* = 0.005) remained independent predictors of higher BCR rate after radical prostatectomy. In the current study, prostate volume was estimated using preoperative TRUS. In the previous literature, prostate size was either measured using preoperative TRUS [17,18] or the prostate weight of radical prostatectomy specimen [19,20]. It has been documented that the prostate weight of surgical specimens correlates with an estimated prostate volume using TRUS [20]. Nevertheless, the association between prostate volume and BCR remains controversial in the previous literature [16,17,18,19,20,21]. While some studies reported an inverse impact of prostate size on BCR [19,20,21], others did not identify a significant prognostic role of prostate size regarding BCR [16,17,18]. However, stratification based on prostate size is highly heterogenous in the previous literature. This heterogeneity renders direct comparisons between different studies impossible. In consequence, further standardization in reporting prostate volume is necessary to draw consistent conclusions regarding the association between prostate volume and BCR. After stratification according to prostate volume ≤40 vs. >40 cm^3^, we did not identify any clinically meaningful or statistically significant differences in pathologic tumor characteristics or adjuvant radiation therapy rates that might explain differences in BCR rates. Furthermore, there are also non-measurable factors that may influence BCR after radical prostatectomy. For example, patients with a large prostate volume may be diagnosed with prostate cancer later than patients with a smaller prostate volume due to the attribution of the increase in PSA serum levels to benign prostatic hyperplasia. This factor may potentially result in a higher tumor volume. Therefore, the consideration of tumor volume percentage in a radical prostatectomy specimen might be another approach to better predict BCR in future analyses [32].

Taken together, in the contemporary study cohort of 821 radical prostatectomy-treated prostate cancer patients of a tertiary care center, a higher preoperative prostate volume strongly predicts BCR rates after radical prostatectomy. These observations remained consistent both when using continuous prostate volume and after categorization based on prostate volume median. Conversely, patient age and BMI do not statistically significantly affect BCR rates within this contemporary study cohort. In consequence, the currently reported observations may indicate that the assessment of preoperative prostate volume could provide additional prognostic value beyond standard pathologic tumor characteristics. Therefore, the consideration of prostate volume might be of additional value in postoperative follow-up.

The present study is not devoid of limitations. First, due to its retrospective nature, a potential for residual selection biases remained, despite systematic adjustment for biases and confounders in multivariable models. This limitation is applicable to all studies relying on a retrospective study design [8,13,14,17]. Second, our study relies on a limited sample size. Third, postoperative follow-up within our study cohort was also limited. In consequence, we cannot exclude the fact that the above-mentioned risk factors may behave differently with longer follow-up. Moreover, other study endpoints that could be equally as interesting as BCR, namely metastasis, cancer-specific, other-cause, or overall mortality, could not be investigated.

## 5. Conclusions

A higher preoperative prostate volume independently predicts BCR after radical prostatectomy, but not age or BMI at surgery. In consequence, patients with elevated prostate volume should be considered for closer postoperative follow-up.

## Figures and Tables

**Table 1:** Descriptive characteristics of 821 prostate cancer patients treated with radical prostatectomy (RP) between January 2014 and June 2023.

Characteristic		Overall n = 821 ^1^
Age (in years)		66 (61, 71)
	≤60	188 (23%)
	61–69	368 (45%)
	≥70	265 (32%)
Body mass index (in kg/m^2^)		26.2 (24.3, 28.8)
	<25.0	280 (34%)
	25.0–29.0	394 (48%)
	≥30.0	147 (18%)
Prostate volume (in cm^3^)		40 (30, 55)
	≤40	430 (52%)
	>40	391 (48%)
D’Amico risk group	high	216 (26%)
	intermediate	485 (59%)
	low	120 (15%)
pTstage	pT2	471 (57%)
	pT3/pT4	350 (43%)
pNstage	pN0	692 (84%)
	pN1	42 (5%)
	pNx	87 (11%)
Gleason Grade Group	1	116 (14%)
	2	425 (52%)
	3	156 (19%)
	4	34 (4%)
	5	90 (11%)
Positive surgical margin	No	563 (69%)
	Yes	237 (29%)
	Unknown	21 (2%)
Robotic-assisted radical prostatectomy		663 (81%)
Nerve-sparing surgery		745 (91%)
Adjuvant radiation therapy		77 (9%)

^1^ Median (interquartile range); n (%). Abbreviations: pNstage = pathologic lymph node stage; pTstage = pathologic tumor stage.

**Table 2:** Univariable and multivariable Cox regression models addressing rates of biochemical recurrence (BCR) after radical prostatectomy (RP), according to patient characteristics (age, body mass index, and prostate volume).

	Univariable			Multivariable Model 1 *			Multivariable Model 2 *		
	HR	95% CI	*p*-Value	HR	95% CI	*p*-Value	HR	95% CI	*p*-Value
Age (continuous)	1.009	0.983, 1.035	0.5	0.991	0.964, 1.019	0.5	–	–	–
61–69 (Ref. ≤ 60 years)	0.74	0.48, 1.15	0.2	–	–	–	0.64	0.41, 1.004	0.1
≥70 (Ref. ≤ 60 years)	1.08	0.70, 1.67	0.7	–	–	–	0.88	0.55, 1.39	0.6
Body mass index (continuous)	1.018	0.976, 1.061	0.4	1.006	0.964, 1.050	0.8	–	–	–
25.0–29.9 (Ref. < 25.0 kg/m^2^)	1.16	0.80, 1.69	0.4	–	–	–	1.10	0.75, 1.63	0.6
≥30.0 (Ref. < 25.0 kg/m^2^)	1.07	0.65, 1.75	0.8	–	–	–	0.88	0.52, 1.48	0.6
Prostate volume (continuous)	**1.010**	1.004, 1.016	**0.002**	**1.012**	1.005, 1.019	**<0.001**	–	–	–
>40 (Ref. ≤ 40 cm^3^)	**1.64**	1.17, 2.31	**0.004**	–	–	–	**1.66**	1.17, 2.36	**0.005**

* Adjusted for PSA, pTstage, Gleason Grade Group, pNstage, positive surgical margin, and adjuvant radiation therapy. Abbreviations: CI = confidence interval; HR = hazard ratio; pNstage = pathologic lymph node stage; PSA = prostate-specific antigen; pTstage = pathologic tumor stage.

## Data Availability

All data generated or analyzed during this study are included in this article. Further enquiries can be directed to the corresponding author.

## References

[1] Williams I.S., McVey A., Perera S., O’brien J.S., Kostos L., Chen K., Siva S., Azad A.A., Murphy D.G., Kasivisvanathan V. (2022). Modern paradigms for prostate cancer detection and management. Med. J. Aust..

[2] Chierigo F., Wenzel M., Würnschimmel C., Flammia R.S., Horlemann B., Tian Z., Saad F., Chun F.K.H., Graefen M., Gallucci M. (2022). Survival after Radical Prostatectomy versus Radiation Therapy in High-Risk and Very High-Risk Prostate Cancer. J. Urol..

[3] Van den Broeck T., van den Bergh R.C., Briers E., Cornford P., Cumberbatch M., Tilki D., De Santis M., Fanti S., Fossati N., Gillessen S. (2020). Biochemical Recurrence in Prostate Cancer: The European Association of Urology Prostate Cancer Guidelines Panel Recommendations. Eur. Urol. Focus..

[4] Pisansky T.M., Thompson I.M., Valicenti R.K., D’Amico A.V., Selvarajah S. (2019). Adjuvant and Salvage Radiotherapy after Prostatectomy: ASTRO/AUA Guideline Amendment 2018–2019. J. Urol..

[5] Tilki D., Chen M.-H., Wu J., Huland H., Graefen M., Wiegel T., Böhmer D., Mohamad O., Cowan J.E., Feng F.Y. (2021). Adjuvant Versus Early Salvage Radiation Therapy for Men at High Risk for Recurrence Following Radical Prostatectomy for Prostate Cancer and the Risk of Death. J. Clin. Oncol..

[6] Cookson M.S., Aus G., Burnett A.L., Canby-Hagino E.D., D’amico A.V., Dmochowski R.R., Eton D.T., Forman J.D., Goldenberg S.L., Hernandez J. (2007). Variation in the Definition of Biochemical Recurrence in Patients Treated for Localized Prostate Cancer: The American Urological Association Prostate Guidelines for Localized Prostate Cancer Update Panel Report and Recommendations for a Standard in the Reporting of Surgical Outcomes. J. Urol..

[7] Würnschimmel C., Wenzel M., Wang N., Tian Z., Karakiewicz P.I., Graefen M., Huland H., Tilki D. (2021). Radical prostatectomy for localized prostate cancer: 20-year oncological outcomes from a German high-volume center. Urol. Oncol. Semin. Orig. Investig..

[8] Preisser F., Chun F.K.H., Pompe R.S., Heinze A., Salomon G., Graefen M., Huland H., Tilki D. (2019). Persistent Prostate-Specific Antigen After Radical Prostatectomy and Its Impact on Oncologic Outcomes. Eur. Urol..

[9] Epstein J.I., Egevad L., Amin M.B., Delahunt B., Srigley J.R., Humphrey P.A., Grading Committee (2016). The 2014 International Society of Urological Pathology (ISUP) Consensus Conference on Gleason Grading of Prostatic Carcinoma: Definition of Grading Patterns and Proposal for a New Grading System. Am. J. Surg. Pathol..

[10] Egevad L., Delahunt B., Srigley J.R., Samaratunga H. (2016). International Society of Urological Pathology (ISUP) grading of prostate cancer—An ISUP consensus on contemporary grading. APMIS.

[11] Wu S., Xie L., Lin S.X., Wirth G.J., Lu M., Zhang Y., Blute M.L., Dahl D.M., Wu C.-L. (2020). Quantification of perineural invasion focus after radical prostatectomy could improve predictive power of recurrence. Hum. Pathol..

[12] Öbek C., Lai S., Sadek S., Civantos F., Soloway M.S. (1999). Age as a prognostic factor for disease recurrence after radical prostatectomy. Urology.

[13] Leyh-Bannurah S.-R., Wagner C., Schuette A., Liakos N., Karagiotis T., Mendrek M., Rachubinski P., Oelke M., Tian Z., Witt J.H. (2022). Feasibility of robot-assisted radical prostatectomy in men at senior age ≥75 years: Perioperative, functional, and oncological outcomes of a high-volume center. Aging Male.

[14] Tilki D., Maurer V., Pompe R.S., Chun F.K., Preisser F., Haese A., Graefen M., Huland H., Mandel P. (2020). Tumor characteristics, oncological and functional outcomes after radical prostatectomy in very young men ≤ 45 years of age. World J. Urol..

[15] Rivera-Izquierdo M., de Rojas J.P., Martínez-Ruiz V., Arrabal-Polo M., Pérez-Gómez B., Jiménez-Moleón J.J. (2022). Obesity and biochemical recurrence in clinically localised prostate cancer: A systematic review and meta-analysis of 86,490 patients. Prostate Cancer Prostatic Dis..

[16] Tzeng M., Vertosick E., Basourakos S.P., Eastham J.A., Ehdaie B., Scardino P.T., Vickers A.J., Hu J.C. (2021). Addition of Prostate Volume and Prostate-specific Antigen Density to Memorial Sloan Kettering Cancer Center Prostate Cancer Nomograms. Eur. Urol. Open Sci..

[17] Mandel P., Weinhold P., Michl U., Huland H., Graefen M., Tilki D. (2015). Impact of prostate volume on oncologic, perioperative, and functional outcomes after radical prostatectomy. Prostate.

[18] Stankovic M., Wolff L. (2024). The Impact of Prostate Volume in Open Radical Prostatectomy: A Single Centre Experience. Clin. Genitourin. Cancer.

[19] Freedland S.J., Isaacs W.B., Platz E.A., Terris M.K., Aronson W.J., Amling C.L., Presti J.C., Kane C.J. (2005). Prostate Size and Risk of High-Grade, Advanced Prostate Cancer and Biochemical Progression After Radical Prostatectomy: A Search Database Study. J. Clin. Oncol..

[20] Jaber A.R., Moschovas M.C., Saikali S., Gamal A., Perera R., Rogers T., Patel E., Sandri M., Tilki D., Patel V. (2024). Impact of Prostate Size on the Functional and Oncological Outcomes of Robot-assisted Radical Prostatectomy. Eur. Urol. Focus..

[21] Kwon T., Jeong I.G., You D., Park M.-C., Hong J.H., Ahn H., Kim C.-S. (2010). Effect of prostate size on pathological outcome and biochemical recurrence after radical prostatectomy for prostate cancer: Is it correlated with serum testosterone level?. BJU Int..

[22] EAU Guidelines Office (2023). EAU Guidelines on Prostate Cancer. Edn. Presented at the EAU Annual Congress Milan 2023.

[23] Regional Office for the Americas of the World Health Organization Overweight and Obesity 2024. https://www.paho.org/en/enlace/overweight-and-obesity.

[24] Hoeh B., Hohenhorst J.L., Wenzel M., Humke C., Preisser F., Wittler C., Brand M., Köllermann J., Steuber T., Graefen M. (2023). Full functional-length urethral sphincter- and neurovascular bundle preservation improves long-term continence rates after robotic-assisted radical prostatectomy. J. Robot. Surg..

[25] Wenzel M., Preisser F., Theissen L.H., Humke C., Welte M.N., Wittler C., Kluth L.A., Karakiewicz P.I., Chun F.K.H., Mandel P. (2020). The Effect of Adverse Patient Characteristics on Perioperative Outcomes in Open and Robot-Assisted Radical Prostatectomy. Front. Surg..

[26] R Core Team (2022). R: A Language and Environment for Statistical Computing. https://www.R-project.org/.

[27] Duwe G., Boehm K., Becker G., Ruckes C., Sparwasser P., Haack M., Dotzauer R., Thomas A., Mager R., Tsaur I. (2024). Individualized center-based analysis of urinary and sexual functional outcome after radical prostatectomy based on the prostate cancer outcome study: A post hoc pathway to patient outcome measurement analysis for quality improvement. World J. Urol..

[28] Kowalski C., Sibert N.T., Hammerer P., Wesselmann S., Feick G., Carl E.-G., Klotz T., Apel H., Dieng S., Nyarangi-Dix J. (2024). Harninkontinenz nach radikaler Prostatektomie beim Prostatakarzinom—*Aktuelle* Daten von 17.149 Patienten aus 125 zertifizierten Zentren. Die Urol..

[29] Langlais C.S., Cowan J.E., Neuhaus J., Kenfield S.A., Van Blarigan E.L., Broering J.M., Cooperberg M.R., Carroll P., Chan J.M. (2019). Obesity at Diagnosis and Prostate Cancer Prognosis and Recurrence Risk Following Primary Treatment by Radical Prostatectomy. Cancer Epidemiol. Biomarkers Prev..

[30] Freedland S.J., Aronson W.J., Kane C.J., Presti J.C., Amling C.L., Elashoff D., Terris M.K. (2004). Impact of Obesity on Biochemical Control After Radical Prostatectomy for Clinically Localized Prostate Cancer: A Report by the Shared Equal Access Regional Cancer Hospital Database Study Group. J. Clin. Oncol..

[31] Maj-Hes A.B., Mathieu R., Özsoy M., Soria F., Moschini M., Abufaraj M., Briganti A., Roupret M., Karakiewicz P.I., Klatte T. (2017). Obesity is associated with biochemical recurrence after radical prostatectomy: A multi-institutional extended validation study. Urol. Oncol. Semin. Orig. Investig..

[32] Alenezi A., Ismail M., Eden C. (2021). Can Tumour Volume Percentage in Radical Prostatectomy Predict Cancer Biochemical Recurrence? Determining a Cut-off Point and Composite Risk Factors Approach. Res. Rep. Urol..

